# Coronary artery bypass grafting in infants and young children: default or alternative choice?

**DOI:** 10.1093/icvts/ivac168

**Published:** 2022-07-01

**Authors:** Bahaaldin Alsoufi

**Affiliations:** Department of Cardiovascular and Thoracic Surgery, Norton Children’s Hospital, University of Louisville School of Medicine, Louisville, KY, USA

**Keywords:** Congenital coronary anomalies, Coronary artery bypass grafting

In the current issue of the *ICVTS*, Hohri and colleagues from Kyoto, Japan report mid-term outcomes of coronary artery bypass grafting (CABG) in 5 young children (age range 6–40 months, including 3 infants) with congenital left main coronary atresia (*n* = 2) or stenosis (*n* = 3). In all cases, the left internal thoracic artery (LITA) was used as a pedicle *insitu* graft to the left anterior descending artery. They report no operative mortality and 100% graft patency at discharge. Graft patency was 80% at median follow-up of 4.2 years, and there were no late deaths or cardiac events. They conclude that CABG in this age group is a useful strategy when needed, although longer follow-up is necessary [1].

Congenital coronary anomalies are well described in children, in isolation or association with other cardiac defects. Nonetheless, contrary to anomalous left coronary origin from the pulmonary artery, for which the indication and surgical strategy are well agreed on, the role of intervention for isolated coronary occlusion in infants and small children is less established [2, 3]. In this age group, symptoms of myocardial ischaemia are often vague, and stress-induced testing for myocardial hypoperfusion is challenging. Due to the rarity of these anomalies, a knowledge gap exists with regards to the implication of coronary occlusion on long-term survival of these children, and whether or not the emerging collaterals are adequate to prevent myocardial ischaemia. Moreover, another knowledge gap exists with regards to the efficacy and durability of surgical revascularization, specially that there is no consensus on optimal surgical management technique for these coronary anomalies.

The more traditional technique to manage proximal coronary stenosis in children is patch angioplasty of the proximal left main coronary artery. This has been well described in children with Williams syndrome, and in some patients with anomalous aortic origin of the coronary artery with intramural course [4, 5]. A similar technique is sometimes employed in neonates with intramural coronaries undergoing the arterial switch operation. It seems that the 3 children in the series from Hohri and colleagues with coronary stenosis could have been candidates for patch angioplasty, and while the literature on long-term outcomes of these procedures in children is sparse and contaminated by the heterogenicity of the patient population, this would have been my choice as a potentially technically less demanding and more tested approach that leaves the potential for future CABG open in the possibility of recurrent obstruction. CABG in children, especially infants and small children as in the study of Hohri and colleagues is technically demanding, and there is always the question about the potential of conduit growth in response to the somatic growth of the children. CABG with LITA to the left anterior descending is the golden choice in adults as it is associated with the best survival and long-term graft patency that can exceed 90% in selected cases [6]. However again, there are little data about graft patency in children and the demand on these grafts in small children is to remain patent for decades longer than that in adults. In adults, competitive flow when the LITA is used on coronaries with moderate stenosis has been associated with decreased flow in the LITA and higher incidence of graft occlusion. In adults, as preoperative proximal coronary stenosis decreased, LITA patency declined [6]. Similarly, in the experience by Hohri and colleagues, one graft became occluded due to competitive flow from the native coronary artery. Survival and graft patency was described in a handful of publications that focused on the paediatric population. A series from France followed 18 children (median age 4 months) who received LITA and or RITA for various coronary pathologies. With a median follow-up of 41 months, there were 2 deaths (1 at least coronary related), 1 occluded graft and 2 percutaneous reinterventions [7]. Another European multi-institutional series followed 80 children (median age 2.3 years) who received different CABG and coronary procedures for various coronary pathologies excluding Kawasaki disease. Hospital mortality was 12/80 (15%) and with a median follow-up of 4 years, 6 patients (8%) needed reintervention [8]. CABG is more commonly performed in children with Kawasaki disease, and longer follow-up has been described in few reports. A series from Japan followed 114 children (median age 10 years) who received CABG with LITA or saphenous vein grafts for Kawasaki disease. Hospital mortality was 0% and with a median follow-up of 19 years, 25-year survival was 95% and cardiac event-free rate was 60%. In addition, 20-year graft patency rate was 87% for LITA and 44% for saphenous vein grafts [9]. Finally, a study examined the paediatric cardiac care consortium and focused on the outcomes of 137 children (median age 6.8 years) who underwent CABG or other coronary revascularizations for congenital coronary anomalies excluding Kawasaki disease. Hospital mortality was 20/137 (15%), mainly in those having rescue operations, and with a median follow-up of 15 years, 15-year transplant-free survival was 91%. In that study, however, graft patency was 58% for CABG although that number might be exaggeratedly low given that angiographic assessments of graft patency were not routinely performed and only in those with clinical suspicion for graft occlusion [10].

In summary, CABG utilizing the LITA can be a useful surgical technique in infants and small children with congenital coronary anomalies. However, given the technical challenges and lack of long-term follow-up, it should be used as an alternative technique rather than first choice. Proximal patch angioplasty might be a more tested technique in the paediatric population that leaves the potential for percutaneous intervention or future CABG open in case of failure.
